# Comparison of the outcomes between sorafenib and lenvatinib as the first-line systemic treatment for HBV-associated hepatocellular carcinoma: a propensity score matching analysis

**DOI:** 10.1186/s12876-022-02210-3

**Published:** 2022-03-25

**Authors:** Na Ryung Choi, Ju Yeon Kim, Ji Hoon Hong, Moon Haeng Hur, Heejin Cho, Min Kyung Park, Jihye Kim, Yun Bin Lee, Eun Ju Cho, Jeong-Hoon Lee, Su Jong Yu, Jung-Hwan Yoon, Yoon Jun Kim

**Affiliations:** 1 Department of Internal Medicine and Liver Research Institute, Seoul National University College of Medicine, Seoul, South Korea; 2 Department of Internal Medicine, Seoul National University Hospital, 101 Daehak-ro, Jongno-gu, Seoul, 03080 Korea

**Keywords:** Hepatitis B, Chronic, Carcinoma, Hepatocellular, Lenvatinib, Sorafenib

## Abstract

**Background/aim:**

In a randomized controlled trial, lenvatinib was non-inferior to sorafenib in overall survival (OS) of patients with unresectable hepatocellular carcinoma (uHCC). This study aimed to compare the effects of sorafenib and lenvatinib as first-line systemic therapy against uHCC with real-world data in chronic hepatitis B patients.

**Methods:**

This retrospective single-center study involved 132 patients with HBV-related uHCC. Propensity score matching (PSM) was used to balance the baseline characteristics, including age, sex, serum alpha-fetoprotein levels, Child–Pugh class, tumor size, and tumor stage. The primary endpoint was overall survival (OS), and the secondary endpoints included progression-free survival (PFS), time to progression (TTP), and tumor response.

**Results:**

After PSM, the final analysis included 44 patients treated with lenvatinib and 88 with sorafenib. The OS (7.0 vs 9.2 months, *p* = 0.070) and PFS (4.6 vs 2.4 months, *p* = 0.134) were comparable between the two drugs. Multivariable analysis showed that lenvatinib and sorafenib were not independent prognostic factors of OS (adjusted hazard ratio = 1.41, 95% confidence interval = 0.96–2.08, *p* = 0.077) after adjustment for baseline alpha-fetoprotein levels, total bilirubin levels, alanine aminotransferase level, performance status, tumor stage, and tumor size. However, the lenvatinib group had a significantly prolonged TTP (5.2 vs 2.5 months, *p* = 0.018) and a higher objective response rate (18.2% vs 4.5%, *p* = 0.020) and disease control rate (77.3% vs 47.7%, *p* = 0.001) than the sorafenib group.

**Conclusions:**

Our study demonstrated that lenvatinib had a comparable OS and PFS but longer TTP and better tumor response compared to sorafenib in patients with HBV-related uHCC.

**Supplementary Information:**

The online version contains supplementary material available at 10.1186/s12876-022-02210-3.

## Introduction

About 3.6% of the world population (over 248 million people) is chronically infected with the hepatitis B virus (HBV). The largest number of chronic HBV patients are in the Western Pacific and African regions (over 95 million and 75 million individuals, respectively); the smallest number of infected people are in the Americas (over 7 million individuals) [1]. A high ecological correlation exists between areas of HBV prevalence and hepatocellular carcinoma (HCC) incidence and mortality worldwide [2]. In several retrospective studies, HBV accounted for 62–75% of HCC [3, 4]. Consequently, chronic hepatitis B is the most common etiology of HCC in Korea [5].

HCC is one of the deadliest malignancies, ranking fourth as a cause of cancer death worldwide [6], and the second in Korea [7]. More than 50% of patients with HCC present with advanced disease at diagnosis [8]. Furthermore, increased survival and better care of patients in earlier stages allow survival until they reach a more advanced stage [9]. Currently, sorafenib and lenvatinib are widely used as a first-line systemic treatment for advanced HCC. Sorafenib is a multikinase inhibitor that inhibits serine/threonine kinases such as Raf-1 and B-Raf, vascular endothelial growth factor (VEGF) receptors 1, 2, and 3, and platelet-derived growth factor receptor β [10, 11]. Lenvatinib inhibits multiple receptor tyrosine kinases such as VEGF receptors 1, 2, and 3, fibroblast growth factor receptors 1, 2, 3, and 4, platelet-derived growth factor receptor β, and RET and KIT oncogenes [12–15].

Randomized controlled phase 3 trials were conducted to prove the efficacy of each drug. After the SHARP trial (2008), sorafenib represented the only systemic treatment with proven efficacy in the treatment of patients with advanced HCC for a decade [16]. In the REFLECT trial (2018), lenvatinib showed improved progression-free survival (PFS) despite similar overall survival (OS) and was approved with sorafenib as a first-line treatment for unresectable HCC (uHCC) [17]. In subgroup analysis of the REFLECT trial, patients with HBV etiology tended to have longer OS as well as better PFS when using lenvatinib compared with sorafenib. Therefore, whether treatment with lenvatinib results in good clinical outcomes such as OS or PFS in patients with HBV-related advanced HCC in real world is questionable.

Additional systemic treatment options are currently available, including the tyrosine kinase inhibitors (TKIs) regorafenib and cabozantinib, the VEGF-receptor inhibitor ramucirumab, and the programmed cell death protein 1 (PD-1) inhibitors nivolumab and pembrolizumab [18]. Patients can receive these treatments as second-line or third-line systemic treatment after first-line treatment. Recently, novel drugs such as combination of atezolizumab and bevacizumab are being studied. However, sorafenib or lenvatinib is used more in practice because both drugs are recommended in the guidelines and are cost-effective [5, 19].

In the present study, the outcomes between sorafenib and lenvatinib as the first-line systemic therapy for HBV-related uHCC were compared using real-world data with propensity score matching (PSM) in terms of OS, PFS, and time to progression (TTP). In addition, tumor response, predictors of tumor progression, and prognostic factors of survival were investigated.

## Materials and methods

### Patients

**Fig. 1:**
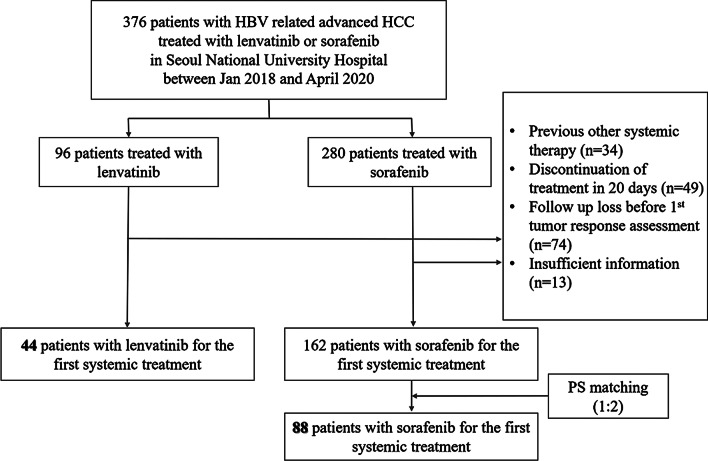
Patient flow. HBV, hepatitis B virus; HCC, hepatocellular carcinoma

This study was a single-center study in Korea. Patients treated with either sorafenib or lenvatinib at a tertiary referral center (Seoul National University Hospital, Seoul, Korea) from January 1, 2018 to April 30, 2020, as a first-line systemic therapy for HBV-related advanced HCC and patients who were not eligible for surgical resection were screened for potential inclusion in the study. For a first-line systemic therapy of advanced HCC, selection of sorafenib or lenvatinib was decided by the clinician because both drugs were suggested in Korean practice guidelines [20]. Patients who received previous local anti-HCC treatments such as radiofrequency ablation (RFA), transcatheter arterial chemoembolization (TACE), transarterial radioembolization (TARE), or hepatic resection, and also liver transplantation prior to sorafenib or lenvatinib treatment were included. Among the 376 eligible patients, patients were excluded if they had received previous systemic treatment (n = 34), discontinued lenvatinib or sorafenib within 20 days after initiation of treatment (n = 49), lost to follow-up before the first tumor response assessment (n = 74), or had insufficient information (n = 13). Finally, 44 patients treated with lenvatinib and 162 patients treated with sorafenib remained after exclusion. Consecutive PSM was conducted by 1:2 (lenvatinib:sorafenib) matching, and finally 44 patients treated with lenvatinib and 88 patients treated with sorafenib were included for statistical analysis as matched cohort (Fig. 1).

### Collected parameters

The following clinical parameters were collected from all 206 patients: (1) demographics including age, sex, Eastern Cooperative Oncology Group (ECOG) performance status, Child–Pugh classification; (2) serum biochemical parameters at baseline including total bilirubin, alanine aminotransferase (ALT), aspartate aminotransferase (AST), prothrombin time (PT), alpha-fetoprotein (AFP), protein induced by vitamin K absence or antagonists-II (PIVKA-II); (3) parameters on tumors including Barcelona Clinic Liver Cancer (BCLC) stage, initial and last follow-up tumor size measured according to the modified Response Evaluation Criteria in Solid Tumor (mRECIST), the presence of extrahepatic metastasis, portal vein invasion, biliary involvement, and previous local treatment of HCC; (4) date of treatment initiation; (5) date of tumor progression; (6) date of death or last follow-up; (7) antiviral drug history; (8) viral status.

### Clinical outcomes and assessment of tumor response

Primary outcome was OS, which was defined as the time interval from the date of initiation of sorafenib or lenvatinib to the date of death or last follow-up. Secondary outcomes were PFS, TTP, and tumor response. PFS was defined as the time from the date of initiation of sorafenib or lenvatinib to the date of radiologic or clinical progression or death from any cause. TTP was defined as the time from the date of initiation of sorafenib or lenvatinib to the date of radiologic or clinical progression. The best tumor response was assessed using the mRECIST. The best tumor response was defined as the best response recorded from the start of sorafenib or lenvatinib treatment until disease progression. Objective response rate (ORR) was defined as complete response (CR) or partial response (PR). Disease control rate (DCR) was defined as CR, PR, or stable disease (SD). Predictive factors associated with OS, PFS and TTP were evaluated, and subgroup analysis was conducted.

### Statistical analysis

Continuous variables were denoted by the median with interquartile range (IQR), and categorical variables were denoted by counts with percentages. Categorical variables were compared using the χ^2^ test. Kaplan–Meier curves were used to estimate OS, PFS and TTP, and the log-rank test was used for group comparisons. The log-rank test was performed with the matched cohort. Hazard ratios (HRs) were estimated using the Cox proportional hazard model. Univariable and multivariable Cox proportional hazard analyses were performed with the unmatched cohort to assess the effects of variables on each outcome of interest. The variables with *p* < 0.05 in the univariable analysis as well as the variable of interest (i.e., lenvatinib treatment (vs. sorafenib)) were used in the multivariable analysis.

To reduce confounding, propensity score was used to match patients treated with lenvatinib to patients treated with sorafenib. The following six covariates expected to be associated with the prognosis of uHCC were considered: age, sex, Child–Pugh classification, BCLC stage, AFP, and tumor size. AFP was stratified into two groups: AFP > 200 ng/mL and AFP ≤ 200 ng/mL. PSM was performed using the nearest-neighbor 1:2 (lenvatinib:sorafenib) matching method with a caliper width of 0.1.

Standardized mean differences (SMDs) were used to evaluate the balance of the observed covariates across groups before and after matching. SMDs were the absolute value of the difference in the mean or proportions divided by the square root of the mean variance [21]. SMDs < 0.25 indicated an acceptable balance of a baseline covariate between the two groups [22].

All statistical tests were two-sided and statistical significance was set at *p* < 0.05. Statistical analysis was performed using SPSS statistics 25.0 (IBM Crop., Armonk, NY, USA) and PSM was performed with R version 4.0.5 (R Foundation for Statistical Computing, Vienna, Austria).

## Results

### Baseline characteristics

Before PSM, a significant imbalance was observed for PT, AFP, Child–Pugh class, ECOG, and subsequent anti-HCC treatment as follows: higher PT international normalized ratio (INR), less frequent AFP > 200 ng/mL, more patients in Child–Pugh class B or C, more patients in ECOG 0, and more patients received subsequent anti-HCC treatment in the lenvatinib group compared to the sorafenib group (Table 1). The final analyses for the primary and secondary outcomes were performed on 44 patients treated with lenvatinib and 88 patients treated with sorafenib (Fig. 1). After PSM, the SMDs of most of the variables were < 0.25, which indicated an acceptable balance between patients treated with lenvatinib or sorafenib after matching (Table 1). Significantly different baseline characteristics were not observed between patients treated with lenvatinib or sorafenib except for subsequent anti-HCC treatment (34.1% vs 52.3%, SMD = − 0.439).

**Table 1:** Baseline characteristics

	Before PSM			After PSM		
	Lenvatinib (n = 44)	Sorafenib (n = 162)	SMD	Lenvatinib (n = 44)	Sorafenib (n = 88)	SMD
Male gender, N (%)	40 (90.9%)	144 (88.9%)	− 0.056	40 (90.9%)	80 (90.9%)	0.000
Age (yr), median (IQR)	58 (51.5–64.8)	59 (53.8–65.0)	− 0.055	58 (51.5–64.8)	58 (52.3–64.8)	− 0.016
ALT (IU/L), median (IQR)	41.5 (25.0–66.0)	34 (25.0–54.0)	0.198	41.5 (25.0–66.0)	37.5 (26.0–65.0)	0.050
AST (IU/L), median (IQR)	52.0 (31.5–89.8)	49 (34.0–78.0)	0.123	52.0 (31.5–89.8)	50.5 (35.3–84.0)	0.029
Total bilirubin (mg/dL), median (IQR)	1.00 (0.60–1.73)	1.00 (0.60–1.30)	0.220	1.00 (0.60–1.73)	1.10 (0.70–1.48)	0.136
Albumin (g/dL), median (IQR)	3.8 (3.2–4.1)	3.7 (3.3–4.1)	− 0.044	3.8 (3.2–4.1)	3.7 (3.1–4.0)	0.099
PT (INR), median (IQR)	1.16 (1.06–1.24)	1.10 (1.03–1.18)	0.256	1.16 (1.06–1.24)	1.10 (1.02–1.20)	0.199
PIVKA (mAU/mL), median (IQR)	1160.0 (115.0–4938.0)	870.5 (110.8–5338.8)	− 0.041	1160.0 (115.0–4938.0)	1174.0 (116.3–4346.8)	0.010
AFP (ng/mL), median (IQR)	132.6 (14.6–5980.0)	427.7 (10.2–4442.5)	− 0.270	132.6 (14.6–5980.0)	86.8 (6.9–3600.0)	− 0.069
*Child–Pugh class, N (%)*			0.277			0.059
A	29 (65.9%)	129 (79.6%)		29 (65.9%)	63 (71.6%)	
B	13 (29.5%)	27 (16.7%)		13 (29.5%)	19 (21.6%)	
C	2 (4.5%)	6 (3.7%)		2 (4.5%)	6 (6.8%)	
*ECOG, N (%)*			− 0.307			− 0.205
0	23 (71.9%)	99 (61.1%)		23 (71.9%)	48 (62.3%)	
1	9 (28.1%)	57 (35.2%)		9 (28.1%)	29 (37.7%)	
*BCLC stage, N (%)*			0.135			− 0.034
B	4 (9.1%)	17 (10.5%)		4 (9.1%)	8 (9.1%)	
C	39 (88.6%)	142 (87.7%)		39 (88.6%)	77 (87.5%)	
D	1 (2.3%)	3 (1.9%)		1 (2.3%)	3 (3.4%)	
Tumor size (mm), median (IQR)	76.5 (40.8–127.5)	58.5 (33.0–124.8)	0.145	76.5 (40.8–127.5)	63.0 (33.0–140.5)	0.062
*Extrahepatic metastasis, N (%)*			0.042			0.075
Yes	31 (70.5%)	111 (68.5%)		31 (70.5%)	59 (67.0%)	
No	13 (29.5%)	51 (31.5%)		13 (29.5%)	29 (33.0%)	
*Portal vein thrombosis, N (%)*			0.120			0.046
Yes	20 (45.5%)	65 (40.1%)		20 (45.5%)	37 (42.0%)	
No	24 (54.5%)	97 (59.9%)		24 (54.5%)	51 (58.0%)	
*Biliary invasion, N (%)*			0.102			0.003
Yes	4 (9.1%)	10 (6.2%)		4 (9.1%)	9 (10.2%)	
No	40 (90.9%)	152 (93.8%)		40 (90.9%)	79 (89.8%)	
*Previous anti-HCC treatment, N (%)*			− 0.109			− 0.056
Yes	35 (79.5%)	136 (84.0%)		35 (79.5%)	72 (81.8%)	
No	9 (20.5%)	26 (16.0%)		9 (20.5%)	16 (18.2%)	
*Subsequent anti-HCC treatment, N (%)*			− 0.536			
Yes	15 (34.1%)	92 (56.8%)		15 (34.1%)	46 (52.3%)	
No	30 (65.9%)	70 (43.2%)		29 (65.9%)	42 (47.7%)	
*Antiviral treatment, N (%)*			0.121			0.107
Yes	35 (79.5%)	122 (75.3%)		35 (79.5%)	67 (76.1%)	
No	8 (18.2%)	37 (22.8%)		8 (18.2%)	20 (22.7%)	
*Viral status*			− 0.083			− 0.067
Immune tolerant phase	0 (0.0%)	0 (0.0%)		0 (0.0%)	0 (0.0%)	
HBeAg +, immune active phase	0 (0.0%)	6 (3.7%)		0 (0.0%)	3 (3.4%)	
Immune inactive phase	35 (79.5%)	118 (72.8%)		35 (79.5%)	63 (71.6%)	
HBeAg−, immune active phase	6 (13.6%)	12 (7.4%)		6 (13.6%)	8 (9.1%)	
HBsAg loss phase	2 (4.5%)	17 (10.5%)		2 (4.5%)	8 (9.1%)	

Data are presented as number (%) or median (interquartile range)

PSM, propensity score matching; SMD, standardized mean difference; ALT, alanine aminotransferase; AST, aspartate aminotransferase; PT, prothrombin time; PIVKA, protein induced by vitamin K absence or antagonists-II; AFP, alpha-fetoprotein; ECOG, Eastern Cooperative Oncology Group; BCLC, Barcelona Clinic Liver Cancer; IQR, inter-quartile range; INR, international normalized ratio

After PSM, the median patient age was 58 years. Most patients (90.9%) were male in both groups. The median PTs were 1.16 INR (IQR, 1.06–1.24 INR) in the lenvatinib group and 1.10 INR (IQR, 1.02–1.20 INR) in the sorafenib group. The median AFPs were 132.6 ng/mL (IQR, 14.6–5980.0 ng/mL) in the lenvatinib group and 86.8 ng/mL (IQR, 6.9–3600.0 ng/mL) in the sorafenib group. The proportions of patients with liver function in Child–Pugh classes A, B, and C were 65.9%, 29.5%, and 4.5%, respectively, in the lenvatinib group and 71.6%, 21.6%, and 6.8%, respectively, in the sorafenib group. The percentages of patients with ECOG 0 and 1 were 71.9% and 28.1%, respectively, in the lenvatinib group and 62.3% and 37.7%, respectively, in the sorafenib group. Fifteen (34.1%) and 46 (52.3%) patients received subsequent anti-HCC treatment including systemic treatment and non-systemic treatment. A total of 35 (75%) and 67 (76.1%) patients received antiviral therapy before initiating lenvatinib or sorafenib, respectively.

Most patients were in the immune-inactive phase of HBV infection at the initiation of either lenvatinib or sorafenib, characterized by hepatitis B e antigen (HBeAg) negativity, hepatitis B e antibody (HBeAb) positivity, persistent normal ALT levels, and HBV DNA levels below 2000 IU/mL [5]. Specifically, 35 (79.5%) patients in the lenvatinib group and 63 (71.6%) in the sorafenib group were in the immune-inactive phase. Patients in the HBeAg-negative immune-active phase made up the second highest number of patients, including 6 (13.6%) patients who received lenvatinib and 8 (9.1%) patients who received sorafenib. The HBeAg-negative immune-active phase is characterized by HBV DNA levels ≥ 2000 IU/mL, increased ALT levels, and HBeAg negativity. In sorafenib group, also 8 (9.1%) patients were in HBsAg loss phase.

### Previous and subsequent anti-HCC treatment and follow-up data

Previous anti-HCC treatment included TACE, TARE, RFA, and surgical resection. Previous anti-HCC treatment was given to 107 patients (81.1%): lenvatinib to 35 patients (79.5%) and sorafenib to 72 patients (81.8%). Subsequent anti-HCC treatment with lenvatinib or sorafenib is presented in Table 2. The patients treated with lenvatinib had less frequent subsequent anti-HCC treatment than patients treated with sorafenib (34.1% vs 52.3%, *p* = 0.048). Most frequent systemic treatment for HCC after first-line lenvatinib was sorafenib (75.0%) and after first-line sorafenib was regorafenib (79.1%).

**Table 2:** Subsequent anti-HCC treatment among patients having received lenvatinib or sorafenib as first systemic treatment

	Lenvatinib (15/44, 34.1%)	Sorafenib (46/88, 52.3%)	*p*-value 0.048
Systemic treatment	12 (80.0%)	43 (93.5%)	
Sorafenib	9 (75.0%)	0 (0.0%)	
Regorafenib	0 (0.0%)	34 (79.1%)	
Nivolumab	0 (0.0%)	6 (14.0%)	
Dendritic cell immunotherapy	1 (8.3%)	0 (0.0%)	
Others	2 (16.7%)	3 (7.0%)	
Locoregional treatment only	3 (20.0%)	3 (6.5%)	

Data are presented as number (%)

We analyzed the common characteristics of 61 patients who received subsequent treatment after first-line systemic therapy. Forty-eight (78.7%) patients were Child–Pugh class A, 42 (68.9%) patients were ECOG 0 or 1, and 39 (63.9%) patients had a tumor size less than 50% of the total liver volume. Thirty-one patients (50.8%) had a liver function of Child–Pugh class A, performance status of ECOG 0 or 1, and tumor size less than 50% of the total liver volume.

At the time of data cut-off, there were 71 deaths (53.8%): 24 in patients treated with lenvatinib (54.5%) and 47 in patients treated with sorafenib (53.4%). Median follow-up was 6.7 months (IQR, 3.0–11.6 months): 4.7 months (IQR, 3.0–9.8 months) for patients treated with lenvatinib and 8.3 months (IQR, 3.0–12.9 months) for patients treated with sorafenib.

### OS and its prognostic factors

**Fig. 2:**
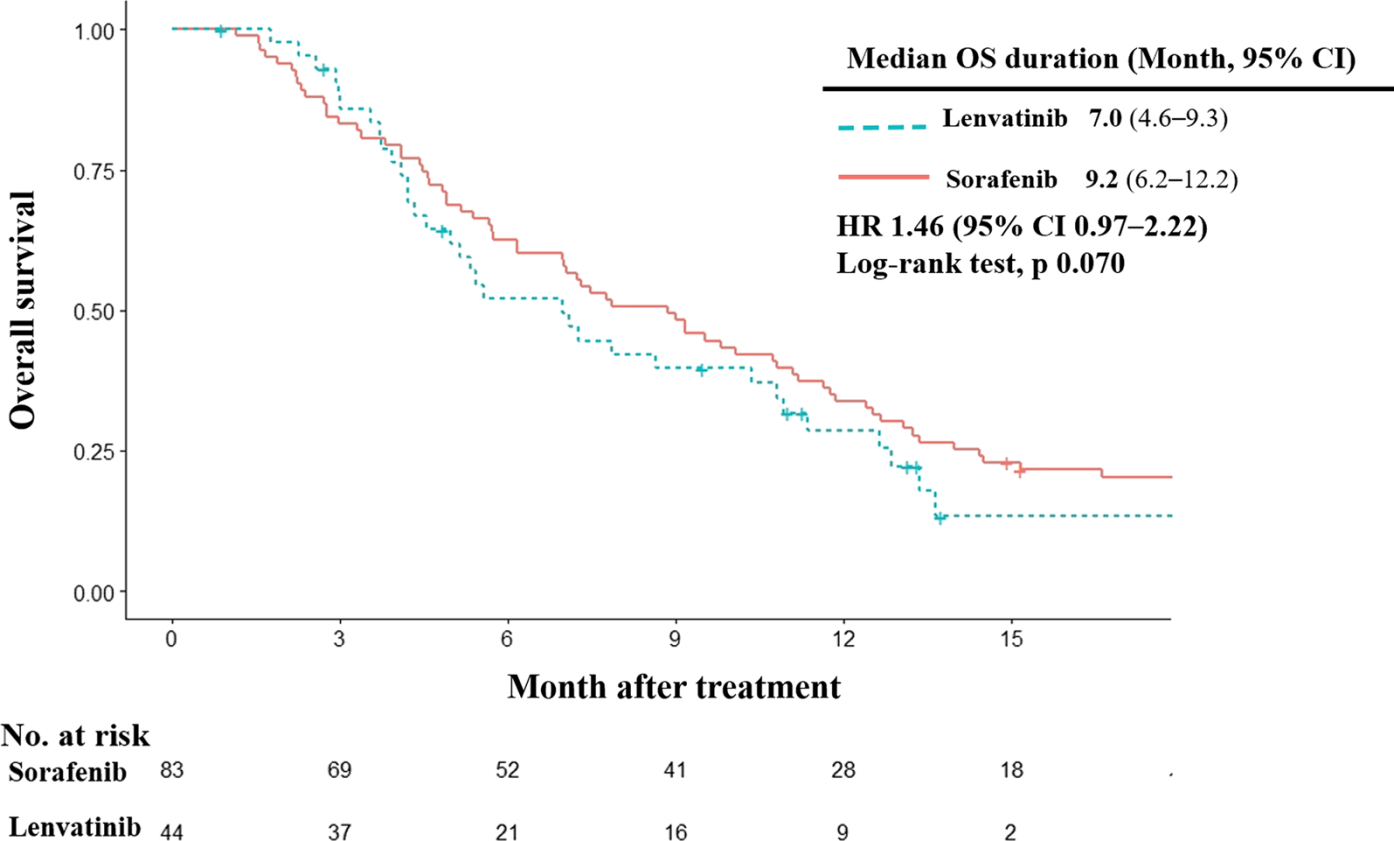
Overall survival. Kaplan–Meier estimates of overall survival by treatment group. OS, overall survival; HR, hazard ratio; CI, confidence interval

Lenvatinib did not show a survival advantage over sorafenib (HR = 1.46; 95% confidence interval, CI 0.97–2.22, log-rank test *p* = 0.070) in patients with HBV-related advanced HCC (Fig. 2). The median survival was 7.0 months in patients treated with lenvatinib (95% CI 4.6–9.3 months) and 9.2 months in patients treated with sorafenib (95% CI 6.2–12.2 months).

To identify prognostic factors of OS, univariable and multivariable analyses were performed with the unmatched cohort (Table 3). The univariable analysis showed that PIVKA-II > 250 mAU/mL, AFP > 200 ng/mL, higher total bilirubin levels, lower serum albumin levels, higher ALT levels, higher AST levels, higher PT INR, the presence of ascites, ECOG 1 (vs 0), Child B or C (vs A), BCLC stage C or D (vs B), larger tumour size, presence of portal vein thrombosis, and previous anti-HCC treatment were significantly associated with poor OS (all *p* < 0.05). From the multivariable analysis for OS, AFP > 200 ng/mL (adjusted HR [aHR] = 1.73, 95% CI 1.23–2.45, *p* = 0.002), higher total bilirubin (aHR = 1.18, 95% CI 1.09–1.29, *p* < 0.001), higher ALT levels (aHR = 1.00, 95% CI 1.00–1.01, *p* = 0.039), ECOG 1 (vs 0) (aHR = 1.98, 95% CI 1.41–2.77, *p* < 0.001), BCLC stage C or D (vs B) (aHR = 1.77, 95% CI 1.01–3.10, *p* = 0.045), and larger tumor size (aHR = 1.01, 95% CI 1.00–1.01, *p* < 0.001) were significant independent prognostic factors associated with the probability of mortality. Based on multivariable analysis, lenvatinib (vs sorafenib) treatment was not an independent risk factor for OS.

**Table 3:** Prognostic factors of OS

	Univariable			Multivariable		
	HR	95% CI	*p-*value	aHR	95% CI	*p-*value
Age > 60 yr	0.78	0.57–1.07	0.125			
Male	1.04	0.63–1.72	0.886			
PIVKA II > 250 mAU/mL	1.77	1.27–2.46	0.001			
AFP > 200 ng/mL	2.11	1.54–2.89	< 0.001	1.73	1.23–2.45	0.002
Total bilirubin	1.20	1.11–1.29	< 0.001	1.18	1.09–1.29	< 0.001
Albumin	0.62	0.47–0.82	0.001			
ALT	1.01	1.00–1.01	0.003	1.00	1.00–1.01	0.039
AST	1.00	1.00–1.01	< 0.001			
PT	2.29	1.40–3.74	0.001			
Presence of ascites	1.97	1.41–2.77	< 0.001			
ECOG 1 (vs 0)	2.09	1.50–2.90	< 0.001	1.98	1.41–2.77	< 0.001
Child B, C (vs A)	1.88	1.32–2.66	< 0.001			
BCLC stage C, D (vs B)	1.72	1.03–2.89	0.039	1.77	1.01–3.10	0.045
Tumor size	1.01	1.01–1.01	< 0.001	1.01	1.00–1.01	< 0.001
Extrahepatic metastasis	0.96	0.69–1.33	0.809			
Portal vein thrombosis	1.53	1.13–2.09	0.007			
Biliary invasion	1.44	0.80–2.60	0.223			
Lenvatinib (vs sorafenib)	1.41	0.96–2.08	0.077	1.36	0.89–2.07	0.154
Previous anti-HCC treatment	0.46	0.31–0.68	< 0.001			

Multivariable analysis was performed using variables with *p* value under 0.05 at univariable analysis

HR, hazard ratio; aHR, adjusted hazard ratio; CI, confidence interval; PIVKA, protein induced by vitamin K absence or antagonists-II; AFP, alpha-fetoprotein; ALT, alanine aminotransferase; AST, aspartate aminotransferase; PT, prothrombin time; ECOG, Eastern Cooperative Oncology Group; BCLC, Barcelona Clinic Liver Cancer

### Secondary outcomes and their predictors

#### PFS

**Fig. 3:**
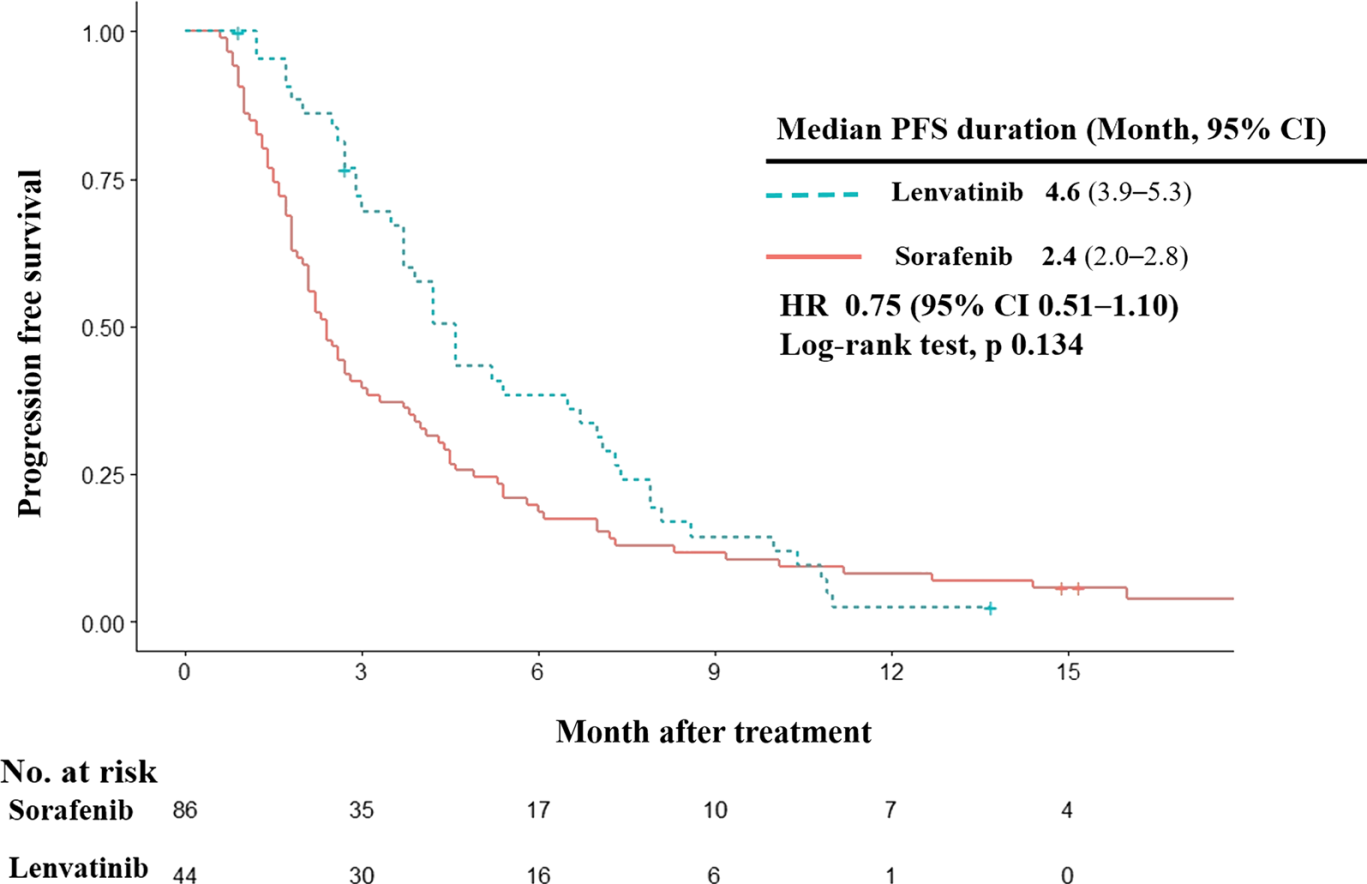
Progression free survival. Kaplan–Meier estimates of progression free survival by treatment group. PFS, progression free survival; HR, hazard ratio; CI, confidence interval

PFS was not prolonged by lenvatinib treatment (HR = 0.75; 95% CI 0.51–1.10, log-rank test *p* = 0.134) in HCC patients with HBV etiology although patients tended to have longer PFS than patients treated with sorafenib (lenvatinib vs sorafenib, 4.6 vs 2.4 months; Fig. 3).

Independent predictors of PFS were studied using univariable and multivariable analyses with the unmatched cohort (Table 4). AFP > 200 ng/mL (HR = 1.79, 95% CI 1.33–2.40, *p* < 0.001) and ECOG 1 (vs 0) (HR = 1.61, 95% CI 1.18–2.21, *p* = 0.003) were significantly associated with shorter PFS. Based on multivariable analysis, lenvatinib (vs sorafenib) treatment was not an independent risk factor for PFS.

**Table 4:** Predictors of PFS

	Univariable			Multivariable		
	HR	95% CI	*p-*value	aHR	95% CI	*p-*value
Age > 60 yr	0.72	0.56–0.99	0.046			
Male	0.81	0.52–1.27	0.364			
PIVKA II > 250 mAU/mL	1.30	0.97–1.75	0.080			
AFP > 200 ng/mL	1.85	1.39–2.47	< 0.001	1.79	1.33–2.40	< 0.001
Total bilirubin	1.08	1.00–1.17	0.063			
Albumin	1.03	0.80–1.34	0.798			
ALT	1.00	1.00–1.01	0.129			
AST	1.00	1.00–1.00	0.183			
PT	1.48	0.92–2.39	0.109			
Presence of ascites	1.23	0.89–1.69	0.212			
ECOG 1 (vs 0)	1.70	1.25–2.33	0.001	1.61	1.18–2.21	0.003
Child B,C (vs A)	1.18	0.85–1.65	0.330			
BCLC stage C,D (vs B)	1.35	0.85–2.14	0.209			
Tumor size	1.00	1.00–1.00	0.060			
Extrahepatic metastasis	1.10	0.81–1.49	0.559			
Portal vein thrombosis	1.07	0.81–1.43	0.626			
Biliary invasion	1.34	0.76–2.36	0.307			
Lenvatinib (vs sorafenib)	0.73	0.52–1.04	0.078	0.77	0.55–1.11	0.159
Previous anti-HCC treatment	0.84	0.58–1.22	0.356			

Multivariable analysis was performed using variables with *p* value under 0.05 at univariable analysis

HR, hazard ratio; aHR, adjusted hazard ratio; CI, confidence interval; PIVKA, protein induced by vitamin K absence or antagonists-II; AFP, alpha-fetoprotein; ALT, alanine aminotransferase; AST, aspartate aminotransferase; PT, prothrombin time; ECOG, Eastern Cooperative Oncology Group; BCLC, Barcelona Clinic Liver Cancer

#### TTP

**Fig. 4:**
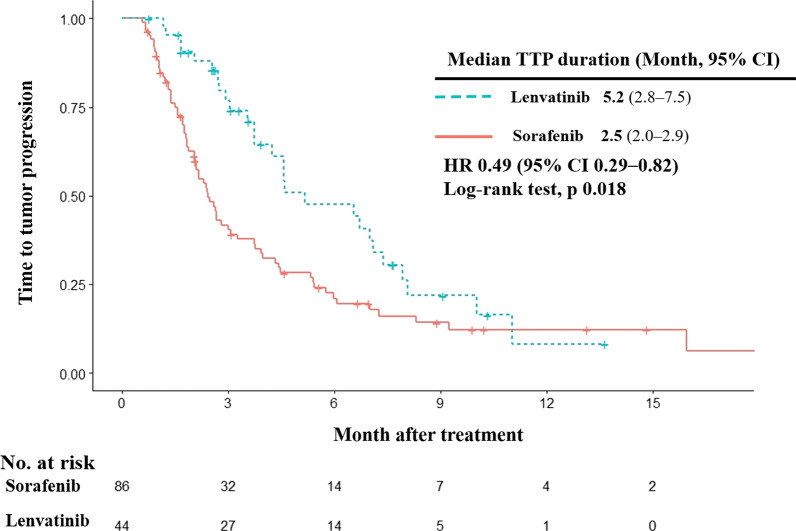
Time to progression. Kaplan–Meier estimates of time to progression by treatment group. TTP, time to progression; HR, hazard ratio; CI, confidence interval

Regarding TTP, patients treated with lenvatinib had significantly longer median TTP than patients treated with sorafenib (HR 0.49; 95% CI 0.29–0.82, log-rank test *p* = 0.018; Fig. 4). Median TTP was 5.2 months (95% CI 2.8–7.5 months) for patients treated with lenvatinib compared with 2.5 months (95% CI 2.0–2.9 months) for patients treated with sorafenib.

In addition, univariable and multivariable analyses were conducted for independent predictors of TTP with the unmatched cohort (Table 5). AFP > 200 ng/mL (HR = 1.98, 95% CI 1.42–2.75, *p* < 0.001) and lenvatinib treatment (vs sorafenib) (HR = 0.55, 95% CI 0.36–0.84, *p* = 0.005) were significant independent predictors of TTP.

**Table 5:** Predictors of TTP

	Univariable			Multivariable		
	HR	95% CI	*p-*value	aHR	95% CI	*p-*value
Age > 60 yr	0.73	0.53–1.00	0.049			
Male	0.75	0.47–1.18	0.213			
PIVKA II > 250 mAU/mL	1.29	0.93–1.80	0.133			
AFP > 200 ng/mL	1.96	1.42–2.72	< 0.001	1.98	1.42–2.75	< 0.001
Total bilirubin	1.02	0.92–1.14	0.663			
Albumin	1.18	0.888–1.59	0.264			
ALT	1.00	1.00–1.00	0.668			
AST	1.00	1.00–1.00	0.236			
PT	1.48	0.88–2.47	0.139			
Presence of ascites	0.97	0.66–1.42	0.867			
ECOG 1 (vs 0)	1.53	1.07–2.17	0.019			
Child B,C (vs A)	0.89	0.60–1.32	0.558			
BCLC stage C,D (vs B)	1.43	0.83–2.48	0.200			
Tumor size	1.00	1.00–1.00	0.828			
Extrahepatic metastasis	1.17	0.83–1.65	0.373			
Portal vein thrombosis	1.05	0.76–1.44	0.787			
Biliary invasion	1.20	0.63–2.28	0.576			
Lenvatinib (vs sorafenib)	0.55	0.37–0.84	0.005	0.55	0.36–0.84	0.005
Previous anti-HCC treatment	0.99	0.63–1.57	0.966			

Multivariable analysis was performed using variables with *p* value under 0.05 at univariable analysis

HR, hazard ratio; aHR, adjusted hazard ratio; CI, confidence interval; PIVKA, protein induced by vitamin K absence or antagonists-II; AFP, alpha-fetoprotein; ALT, alanine aminotransferase; AST, aspartate aminotransferase; PT, prothrombin time; ECOG, Eastern Cooperative Oncology Group; BCLC, Barcelona Clinic Liver Cancer

#### Tumor response

When evaluating tumor response using mRECIST in patients treated with lenvatinib, CR was observed in 2 (4.5%), PR in 6 (13.6%), SD in 26 (59.1%), and PD in 10 (22.7%; Table 6) patients. In patients treated with sorafenib, CR was observed in 3 (3.4%), PR in 1 (1.1%), SD in 38 (43.2%), and PD in 44 (50.0%) patients. Therefore, lenvatinib had a better ORR (18.2% vs 4.5%, *p* = 0.020) and DCR (77.3% vs 47.7%, *p* = 0.001) than sorafenib.

**Table 6:** Tumor response

	Lenvatinib (N, %)	Sorafenib (N, %)	*p*-value
CR	2 (4.5%)	3 (3.4%)	
PR	6 (13.6%)	1 (1.1%)	
SD	26 (59.1%)	38 (43.2%)	
PD	10 (22.7%)	44 (50.0%)	
Not assessed	0 (0%)	2 (2.3%)	
ORR	18.2%	4.5%	0.020
DCR	77.3%	47.7%	0.001

Data are presented as number (%)

CR, complete response; PR, partial response; SD, stable disease; PD, progressive disease; ORR, objective response rate; DCR, disease control rate

### Subgroup analysis for OS, PFS, TTP

**Fig. 5:**
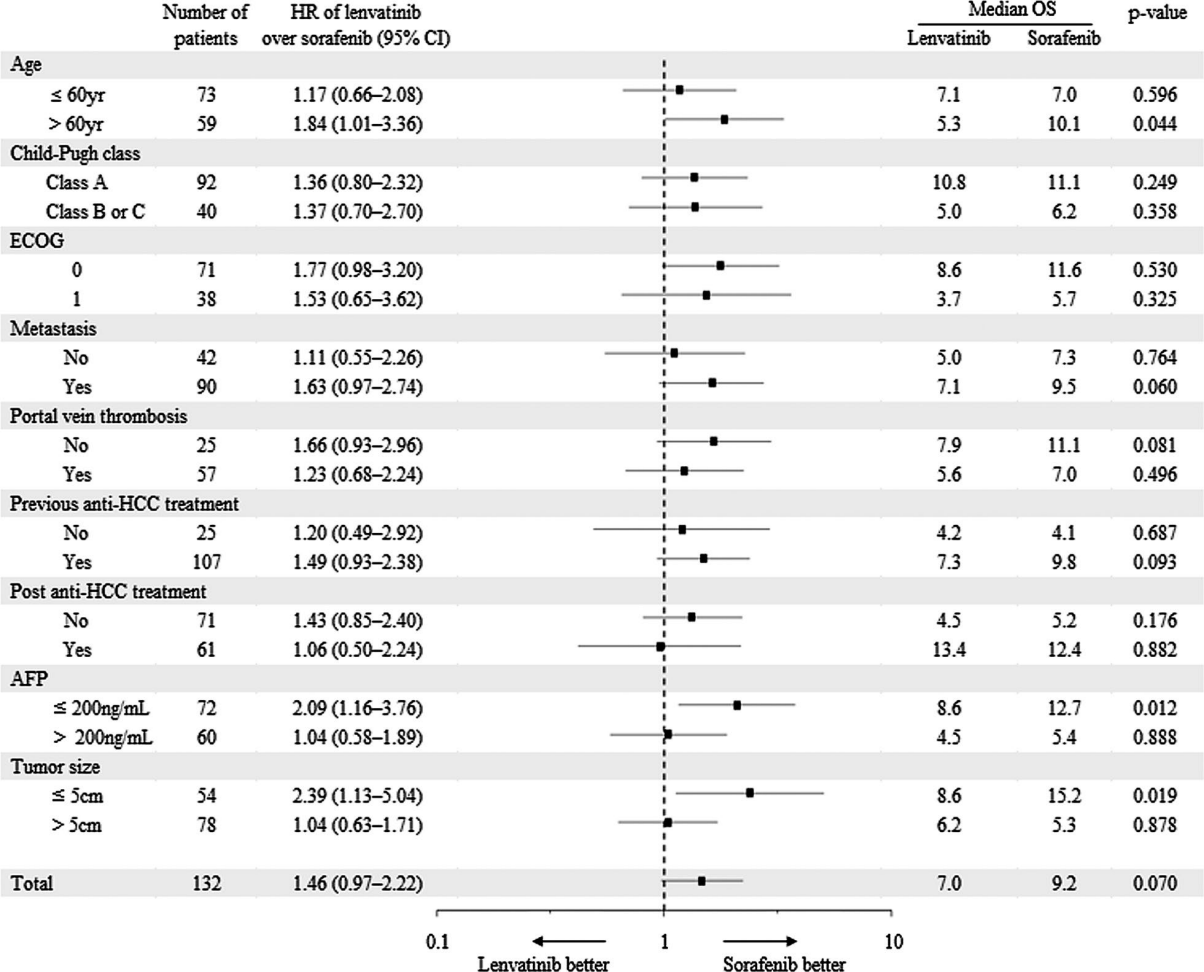
Forest plot of overall survival. Subgroup analysis for overall survival. OS, overall survival; HR, hazard ratio; ECOG, Eastern Cooperative Oncology Group; AFP, alpha-fetoprotein

Subgroup analysis was performed to investigate characteristics of patients who benefited from lenvatinib or sorafenib treatment. Each variable was stratified as shown in Fig. 5. Sex, BCLC stage, and biliary invasion were excluded in subgroup analysis because the number of patients in the subdivided group was too small to conduct Kaplan–Meier estimate: female (n = 12); BCLC B (n = 12); presence of biliary invasion (n = 8). Sorafenib provided longer survival than lenvatinib in patients> 60 years of age (HR = 1.84, 95% CI 1.01–3.36, *p* = 0.044), AFP ≤ 200 ng/mL (HR = 2.09, 95% CI 1.16–3.76, *p* = 0.012), and tumor size ≤ 5 cm (HR = 2.39, 95% CI 1.13–5.04, *p* = 0.019; Fig. 5).

**Fig. 6:**
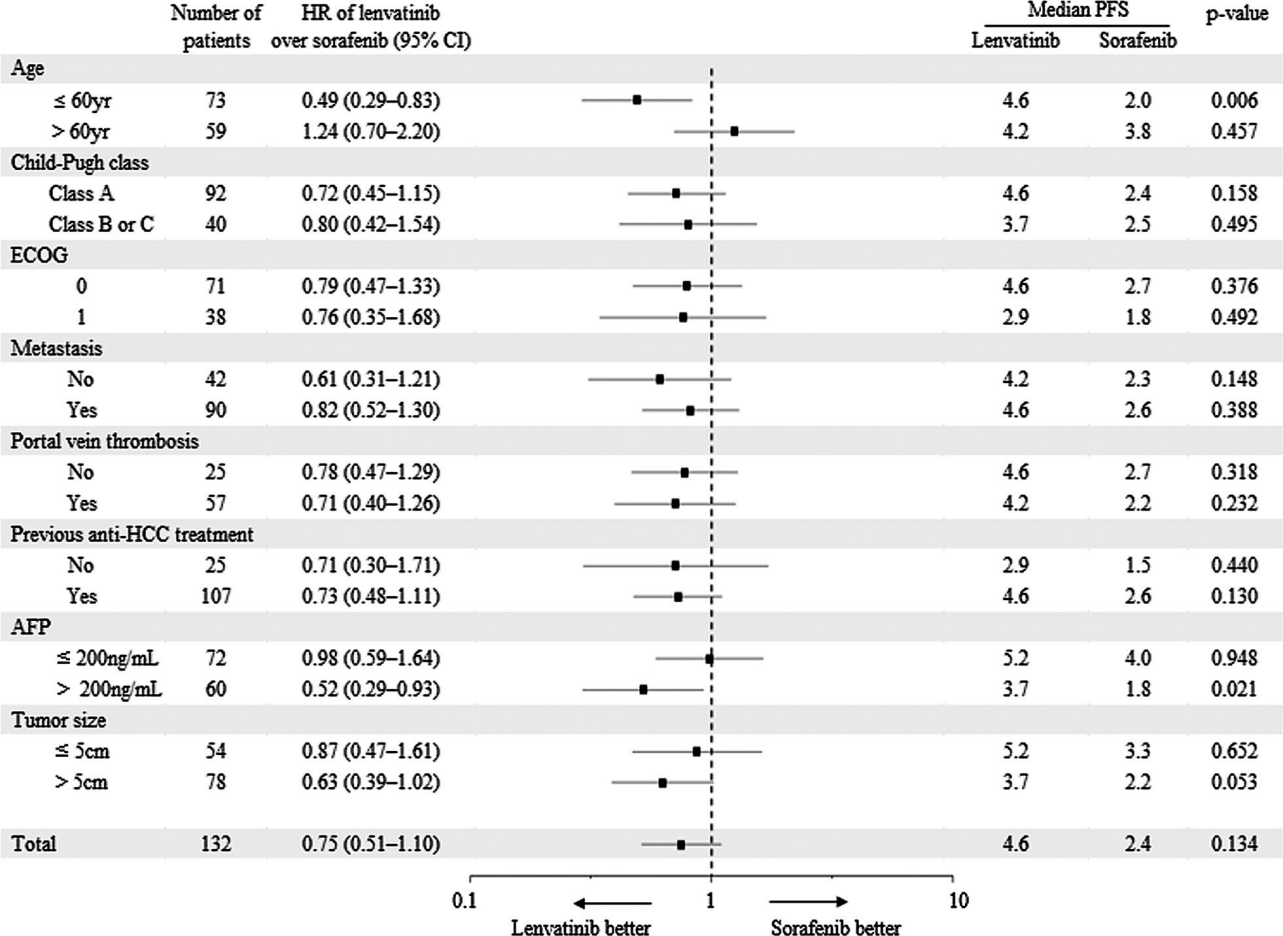
Forest plot of progression free survival. Subgroup analysis for progression free survival. PFS, progression free survival; HR, hazard ratio; ECOG, Eastern Cooperative Oncology Group; AFP, alpha-fetoprotein

In contrast, in subgroup analysis for PFS, lenvatinib showed longer median PFS in patients ≤ 60 years of age (HR = 0.49, 95% CI 0.29–0.83, *p* = 0.006) and AFP > 200 ng/mL (HR = 0.52, 95% CI 0.29–0.93, *p* = 0.021; Fig. 6).

**Fig. 7:**
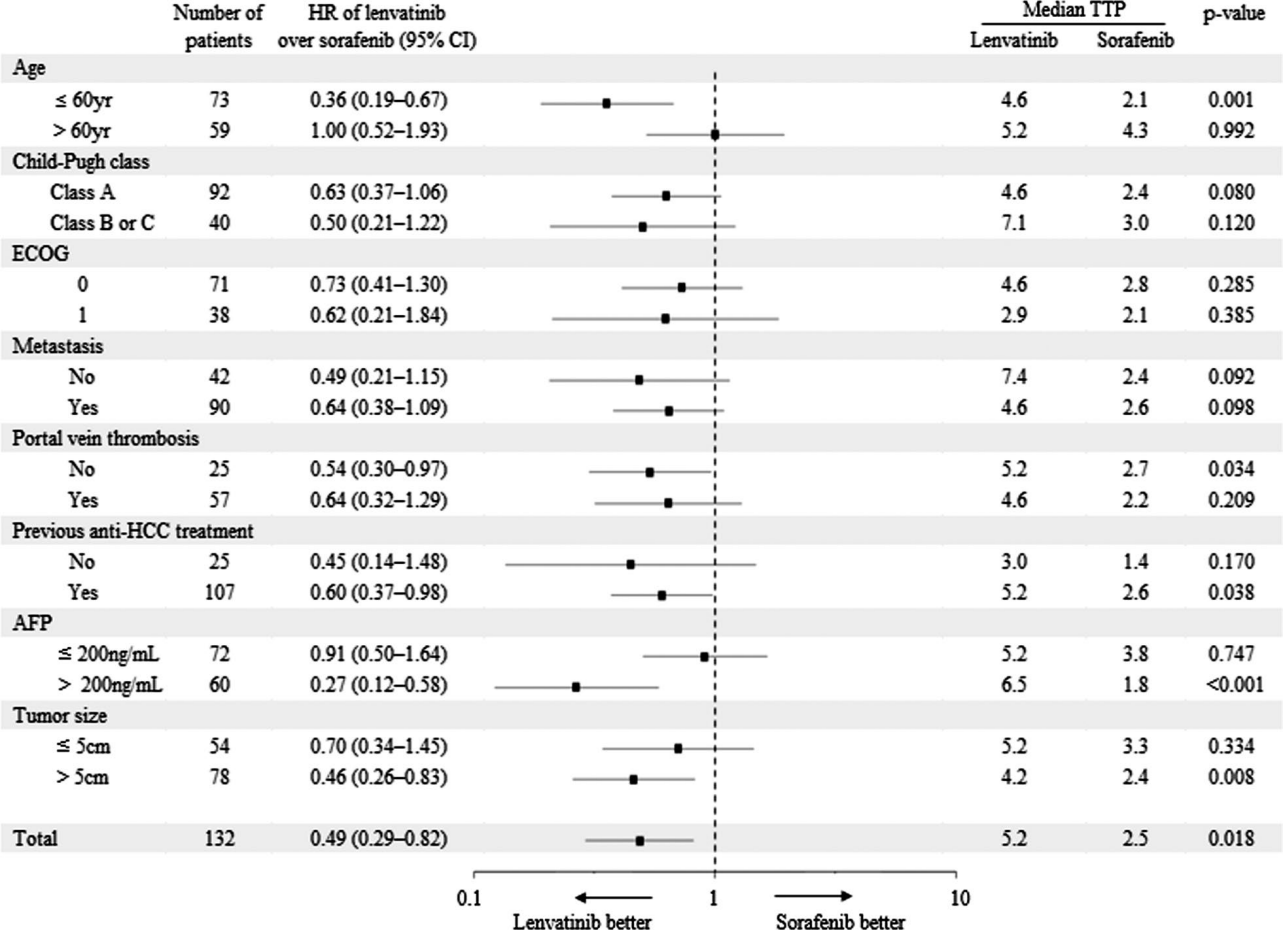
Forest plot of time to progression. Subgroup analysis for time to progression. TTP, time to progression; HR, hazard ratio; ECOG, Eastern Cooperative Oncology Group; AFP, alpha-fetoprotein

Lenvatinib showed longer median TTP than sorafenib in patients ≤ 60 years of age (HR = 0.36, 95% CI 0.19–0.67, *p* = 0.001), without portal vein thrombosis (HR = 0.54, 95% CI 0.30–0.97, *p* = 0.034), with previous anti-HCC treatment (HR = 0.60, 95% CI 0.37–0.98, *p* = 0.038), AFP > 200 ng/mL (HR = 0.27, 95% CI 0.12–0.58, *p* < 0.001), and tumor size > 5 cm (HR = 0.46, 95% CI 0.26–0.83, *p* = 0.008; Fig. 7).

Additional analysis was performed by dividing the lenvatinib and sorafenib groups into subgroups (with or without subsequent treatment; Additional file 1: Figure S1). Patients who received subsequent treatment after first-line sorafenib (Group 1) had longer OS than patients without subsequent treatment after first-line sorafenib treatment (Group 3) (12.4 vs 5.2 months, *p* = 0.004), and than those without subsequent treatment after first-line lenvatinib (Group 4) (12.4 vs 4.5 months, *p* < 0.001). Patients who received subsequent treatment after first-line lenvatinib (Group 2) had longer OS than patients without subsequent treatment after first-line levatinib treatment (Group 4) (13.4 vs 4.5 months, *p* = 0.001), and than those without subsequent treatment after first-line sorafenib (Group 3) (13.4 vs 5.2 months, *p* = 0.029). There is no significant difference in OS between groups with subsequent treatment after sorafenib (Group 1) and lenvatinib (Group 2) (12.4 vs 13.4 months, *p* = 0.882), and between groups without subsequent treatment after sorafenib (Group 3) and lenvatinib (Group 4) (5.2 vs 4.5 months, *p* = 0.176).

In short, The OS of the subgroups receiving subsequent treatment was longer than those without subsequent treatment regardless of the first-line medication. The sorafenib and lenvatinib subgroups that received subsequent treatment had comparable OS, and both subgroups without subsequent treatment had similar OS.

## Discussion

In the present study, 44 HBV-related uHCC patients received lenvatinib and 88 received sorafenib after PSM; lenvatinib treatment did not show a prolonged OS compared to sorafenib (7.0 vs 9.2 months, *p* = 0.070). Median PFS was also not prolonged in patients treated with lenvatinib (4.6 vs 2.4 months, *p* = 0.134). However, TTP in patients treated with lenvatinib was significantly longer than in patients treated with sorafenib (5.2 vs 2.5 months, *p* = 0.018). Furthermore, ORR and DCR were higher in patients treated with lenvatinib than in patients treated with sorafenib.

Subgroup analysis for OS in the REFLECT international Phase 3 trial had a lower HR for lenvatinib than sorafenib in HBV etiology compared with other etiologies [17]. Lenvatinib showed potential effectiveness in prolonging OS of HBV-related uHCC patients in that study. Thus, OS, PFS, TTP and other clinical outcomes were compared between HBV-related uHCC patients treated with lenvatinib or sorafenib in the present study using a real-world cohort. In our study, lenvatinib did not prolong OS longer than sorafenib in chronic hepatitis B patients.

In previous retrospective studies, lenvatinib and sorafenib were also compared in a real-world setting [23]. However, the baseline characteristics of the patients treated with lenvatinib or sorafenib in the previous study differed significantly in several variables including age, PLT count, PIVKA-II, portal vein invasion, and previous anti-HCC treatments. In the present study, based on PSM matching, the baseline characteristics were comparable between the two treatments except for subsequent anti-HCC treatment. Unlike the previous study, only chronic hepatitis B patients were included in the present study [24], and we analyzed various clinical outcomes, including OS, PFS, TTP, tumor response, prognostic factors of OS, predictors of PFS and TTP, and subgroup analyses in detail. The most common etiology of HCC in Korea is HBV [5], and HBV replication can be effectively suppressed by antiviral treatment, which may attenuate the progression of the underlying liver disease that may affect survival. Thus, we included only homogeneous HBV-related HCC patients in this study. To our knowledge, this study is the first real-world study comparing lenvatinib and sorafenib among HBV related uHCC patients.

Median OS, PFS, and TTP for both treatments were shorter in the present study than in the REFLECT trial. This discrepancy may be due to different inclusion criteria between studies. The present study inclusion criteria were extended to patients with Child class B or C (n = 40, 30.3%), with 50% or higher liver occupation (n = 67, 50.8%), with biliary invasion (n = 13, 9.8%), or invasion of main portal vein (n = 17, 12.9%). The inclusion criteria of the present study were extended because lenvatinib was likely to be administered to other patients in addition to only those recommended in the guidelines and the inclusion criteria of the REFLECT trial in the real-world clinic. Therefore, the expanded inclusion criteria in the present study might reflect a more real-world situation.

Lenvatinib demonstrated higher ORR and DCR and longer TTP, but a similar OS compared to sorafenib in HBV-related uHCC patients. These results could mean that patients in whom tumor progression was delayed more with lenvatinib tended to have a relatively shorter time to death than with sorafenib treatment. This finding might be attributed to the subsequent anti-HCC treatment. Patients with post-lenvatinib or post-sorafenib treatment had significantly longer OS than those who did not receive subsequent anti-HCC treatment after lenvatinib or sorafenib (Additional file 1: Figure S1). There are more patients who received subsequent treatment in sorafenib group than in the lenvatinib group (Table 2). Therefore, although the first-line lenvatinib treatment was more effective than sorafenib to have higher ORR and DCR and prolonged TTP, more frequent subsequent anti-HCC treatment after the first-line treatment in the sorafenib group led comparable OS with the lenvatinib group.

The characteristics of patients who received subsequent treatment after the first-line medication included preserved liver function (Child–Pugh A), good performance status (ECOG 0 or 1), and low tumor burden (tumor size < 50% of total liver volume). Patients with Child–Pugh A liver function made up the majority of the patients receiving subsequent treatment. Because more of the patients receiving subsequent treatment had the first-line sorafenib, it might suggest there were more patients whose liver function was preserved in the sorafenib group than in the lenvatinib group. Even though the tumor progressed, preserving liver function could be an important factor in enabling subsequent treatment.

According to previous studies, 39% of patients had decreased liver function from Child–Pugh A to B after lenvatinib treatment [25] as indicated by increased albumin-bilirubin (ALBI) grade [26]. However, there is a lack of research examining liver function after sorafenib treatment except for some case reports of liver dysfunction [27, 28].

Another reason why the sorafenib group had more patients to receive subsequent treatment might be related to the insurance system for second-line treatment. Unlike sorafenib, there is no evidence for the effectiveness of molecularly targeted agents in advanced HCC after progression following lenvatinib treatment [29]. However, sequential treatment with sorafenib-regorafenib is covered by the National Health Insurance System of Korea after RESORCE trial, and in a previous study, 26-month survival was reportedly possible [30]. In the REFLECT trial, HBV etiology likely prolonged OS with lenvatinib treatment; however, in the real world, a lack of subsequent anti-HCC treatment after lenvatinib produced a similar OS to patients treated with sorafenib.

Notably, in our study, lenvatinib was preferred in subgroups for TTP and PFS, and sorafenib was preferred in subgroups for OS. The lenvatinib-preferred subgroups for TTP or PFS and the sorafenib-preferred subgroups for OS did not overlap. Lenvatinib was preferred for patients ≤ 60 years of age, AFP > 200 ng/mL, tumor size > 5 cm, without portal vein thrombosis, with previous anti-HCC treatment for TTP, and was preferred for patients ≤ 60 years of age and AFP > 200 ng/mL for PFS. Sorafenib was preferred in patients > 60 years of age, AFP ≤ 200 ng/mL, and tumor size ≤ 5 cm for OS. These results indicate that lenvatinib might be more effective in suppressing tumor progression in younger patients with high AFP levels, and sorafenib and post-sorafenib anti-HCC treatment might be more effective in the elderly with low AFP levels and small tumor size. It seems that lenvatinib is effective against more aggressive tumors (AFP > 200 ng/mL and tumor size > 5 cm), and post-sorafenib treatment is effective with a lower tumor burden (AFP ≤ 200 ng/mL and tumor size ≤ 5 cm).

In the REFLECT trial [17], lenvatinib (57%) tended to have more patients than sorafenib (49%) who had treatment-related and treatment-emergent adverse events of grade 3 or higher according to the National Cancer Institute Common Terminology Criteria for Adverse Events. Lenvatinib (18%) also tended to have more serious treatment-related and treatment-emergent adverse events than sorafenib (10%). In another study of lenvatinib for thyroid cancer, patients who were older than 65 years had a higher percentage of grade 3 toxicity than those younger than 65 years (89% vs 67%) [31]. Therefore, it seems that younger patients (≤ 60 years of age) had more tolerability to the toxic effects of lenvatinib. In the older group, the risk of toxicity might have been greater than the benefit of anti-tumor effect with lenvatinib. Considering a low tendency of sorafenib to have severe toxicity, sorafenib might be relatively preferred in older groups (> 60 years of age). These observations could suggest further clinical studies in which lenvatinib and sorafenib could have different efficacies in different conditions for uHCC patients associated with HBV infection.

The present study had several strengths. The focus was on the most common etiology of HCC, namely HBV. Subgroup analyses were performed using data from patients with HBV-associated HCC as well as OS, PFS, TTP, and tumor response. Second, PSM was performed to achieve balance between baseline characteristics of the patients treated with lenvatinib or sorafenib. Because selection of the treatment depended on clinician decision, selection bias likely existed. After PSM, the observed variables at baseline that resulted in group differences were removed to limit selection bias as much as possible. Third, the inclusion criteria were broad, thus, data analysis was performed of patients who were in a markedly advanced stage and had no other treatment choice than systemic treatment. In actual clinical practice, many advanced HCC patients receive systemic treatment beyond the guidelines. Therefore, the present study reflected actual clinical situations.

The present study had several limitations. First, this was a retrospective and nonrandomized study. Second, the study was conducted at a single institution and included a small sample size. Therefore, additional studies with a larger number of patients are required to confirm our findings. In addition, the subsequent agents of lenvatinib need to be further studied to obtain better clinical outcomes.

## Conclusions

In conclusion, in real-world data of advanced HBV-related HCC with PSM, lenvatinib showed better TTP, ORR, and DCR than sorafenib but was comparable with sorafenib in terms of OS and PFS. In subgroup analyses, our results showed OS could be prolonged with subsequent treatment after first-line lenvatinib or sorafenib in HBV-related HCC patients.

## Supplementary Information

**Additional file 1. Figure S1.** Overall survival of four subgroups divided by first-line medication and subsequent treatment.

## Data Availability

The datasets used and analyzed during the current study are available from the corresponding author on reasonable request.

## Abbreviations

AFP: Alpha-fetoprotein
ALT: Alanine aminotransferase
AST: Aspartate aminotransferase
BCLC: Barcelona Clinic Liver Cancer
CR: Complete response
DCR: Disease control rate
ECOG: Eastern Cooperative Oncology Group
HBV: Hepatitis B virus
HCC: Hepatocellular carcinoma
INR: International normalized ratio
IQR: Interquartile range
ORR: Objective response rate
PD: Progressive disease
PIVKA: Protein induced by vitamin K absence or antagonists-II
PR: Partial response
PT: Prothrombin time
SD: Stable disease
uHCC: Unresectable hepatocellular carcinoma
