# Supplementary material for: Comparison of the outcomes between sorafenib and lenvatinib as the first-line systemic treatment for HBV-associated hepatocellular carcinoma: a propensity score matching analysis
Source: BMC Gastroenterol. 2022 Mar 25;22:135. doi: 10.1186/s12876-022-02210-3 (PMC8951695; doi:10.1186/s12876-022-02210-3)
Supplement: Supplementary file 1 — Additional file 1. Figure S1. Overall survival of four subgroups divided by first-line medication and subsequent treatment. [file 12876_2022_2210_MOESM1_ESM.docx]

**Additional file 1: Figure S1 Legend**

**Additional file 1: Figure S1. Overall survival of four subgroups divided by first-line medication and subsequent treatment**

Kaplan Meier survival curves of patients with and without subsequent treatment after administration of lenvatinib or sorafenib.

**Additional file 1: Figure S1. Overall survival of four subgroups divided by first-line medication and subsequent treatment**


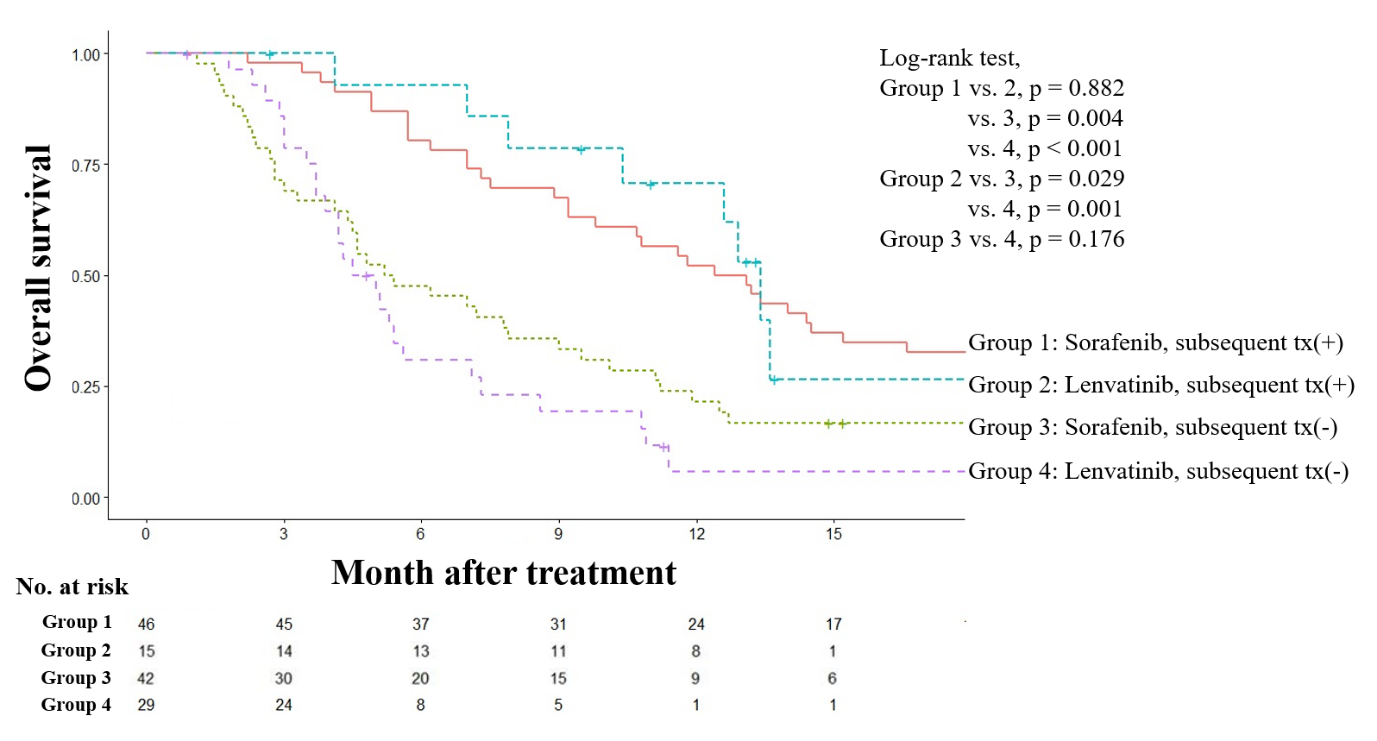


Kaplan Meier survival curves of patients with and without subsequent treatment after administration of lenvatinib or sorafenib.
